# A huge staghorn renal stone: Is there still a need for open surgery to protect against further damage to the kidney? A case report

**DOI:** 10.5339/qmj.2023.30

**Published:** 2023-10-25

**Authors:** Dharmendra Kumar Pipal, Saurabh Jain, Prakash Biswas

**Affiliations:** ^1^Department of General Surgery, All India Institute of Medical Sciences, Gorakhpur, Uttar Pradesh, India Email: dr.dharmendrapipal2007@gmail.com ORCID iD: https://orcid.org/0000-0002-4162-8617; ^2^Arihant Hospital, Bhilwara, Rajasthan, India

**Keywords:** staghorn calculus, percutaneous nephrolithotomy, open stone surgery, extracorporeal shock wave lithotripsy, retrograde intrarenal surgery

## Abstract

Introduction: Staghorn calculi (SC) occupy the renal pelvis and calyces and are common in females linked to repeated urinary tract infections (UTIs). Judicious surgery planning reduces the chance of further damage to the kidney due to renal SCs. Open stone surgery (OSS) is one of the various operative techniques to remove such huge stones with one operative intervention and protect the kidney from ongoing functional damage.

Case Report: A 47-year-old male patient presented with right-sided renal colic pain, and on further investigations, he was diagnosed with a large renal stone responsible for substantial renal function impairment on the same side. The SC measured 8 × 4 cm with another stone in the lower calyx. thinner parenchyma, and only 16% relative function. Therefore, open surgery was selected over less invasive approaches because multiple lithotripsy (ESWL) sittings may have been required in less invasive options.

Discussion: SCs, which can be complete or partial, often result in renal impairment. Hence, it is crucial to implement a proactive therapeutic approach that includes a thorough evaluation of the stone’s size and position, the patient’s choice, and institutional capacity. Complete elimination of SCs is preferred to maintain maximal renal function. Based on clinical, technical, and socioeconomic considerations, open pyelolithotomy or OSS was chosen over percutaneous nephrolithotomy for SC removal in the discussed case.

Conclusion: The ability to remove large stones in a single intervention with open pyelolithotomy has been very effective due to its distinctive clinical presentation and pathological abnormalities.

## Abbreviations

SC: staghorn calculus; PCNL: percutaneous nephrolithotomy; ESWL: extracorporeal shock wave lithotripsy; OSS: open stone surgery; UC: urinary calculus; USS KUB: ultrasonography kidney ureter and urinary bladder; NCCT: non-contrast CT scan; UTI: urinary tract infection; SFR: stone-free rate.

## Introduction

A common urologic disease seen in the emergency room is urinary calculus. Urinary calculi (UC) of the type known as “staghorn calculi” (SCs) are found in the renal collecting system, notably in the renal pelvis and at least two renal calyces. Struvite stones, mostly made of magnesium ammonium phosphate, are commonly associated with persistent urinary tract infections (UTIs) driven by microorganisms that produce urease.^1^ Gram-positive and Gram-negative bacteria, including *Proteus, Staphylococcus, Pseudomonas, Providencia*, and *Klebsiella*, are linked to the development of struvite stones. These bacteria generate the enzyme urease. However, not all strains generate the enzyme that splits urea. While all *Proteus*, Providencia, and Morganella species may manufacture urease, not all Klebsiella and *Staphylococcus* species can.^1,2^ Struvite stones constitute 10%-15% of urinary calculi in developing countries, with a greater prevalence among females than males.^1^ Prompt diagnosis and management in developed countries result in a lower incidence of renal stones.^2^ While unilateral presentation is frequent, bilateral involvement can occur in around 15% of cases.^33^ Struvite stones are associated with various risk factors such as female gender, age extremes, congenital urinary tract malformations, urinary stasis and diversion, neurogenic dysfunction, long-term indwelling Foley catheters, distal renal tubular acidosis, medullary sponge kidney, and diabetes mellitus.^1,2^ This condition often leads to urosepsis and renal failure. Previous reports have linked SCs with increased morbidity and mortality rates.^4^ Open stone surgery (OSS) has been the conventional treatment for a significant period for kidney stones associated with ureteropelvic junction obstruction (UPJO). It can address concurrent anatomical defects and improve renal function. Although there are several procedures available for treating complex calculi, including extracorporeal shock wave lithotripsy (ESWL) using shock wave extracorporeally, PCNL by the percutaneous route, and fragmentation of stone intracorporeally under image guidance, ureterorenoscopy and stone removal by dormia basket and forceps or sometimes evaporation of stone by holmium laser.^4–6^ PCNL is a frequently employed method for extracting sizable SCs, but it entails potential hazards such as pelvicalyceal stem disruption, intestinal perforation, and bleeding.^6^ The unusual case of an unexpectedly large SC presented to the Department of Surgery, All India Institute of Medical Sciences, Gorakhpur, India, and how it was successfully treated utilizing the OSS approach is described in this paper. Now, with the patient’s signed informed consent and institutional approval, it is presented for publication.

## Case Report

**Figure 1.**
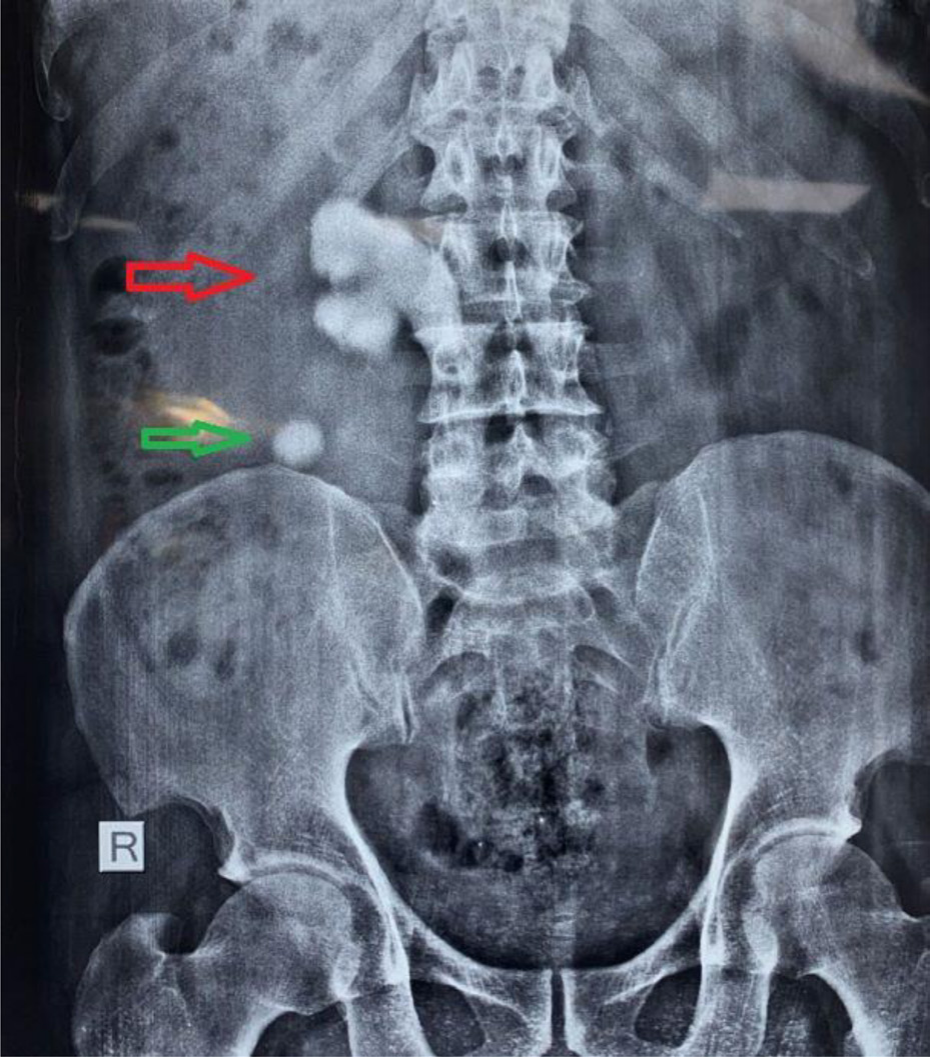
Plain x-ray KUB showing a huge renal pelvic stone extending to the upper ureter and another small stone in the lower calyx, depicted by red and green arrows, respectively.

**Figure 2.**
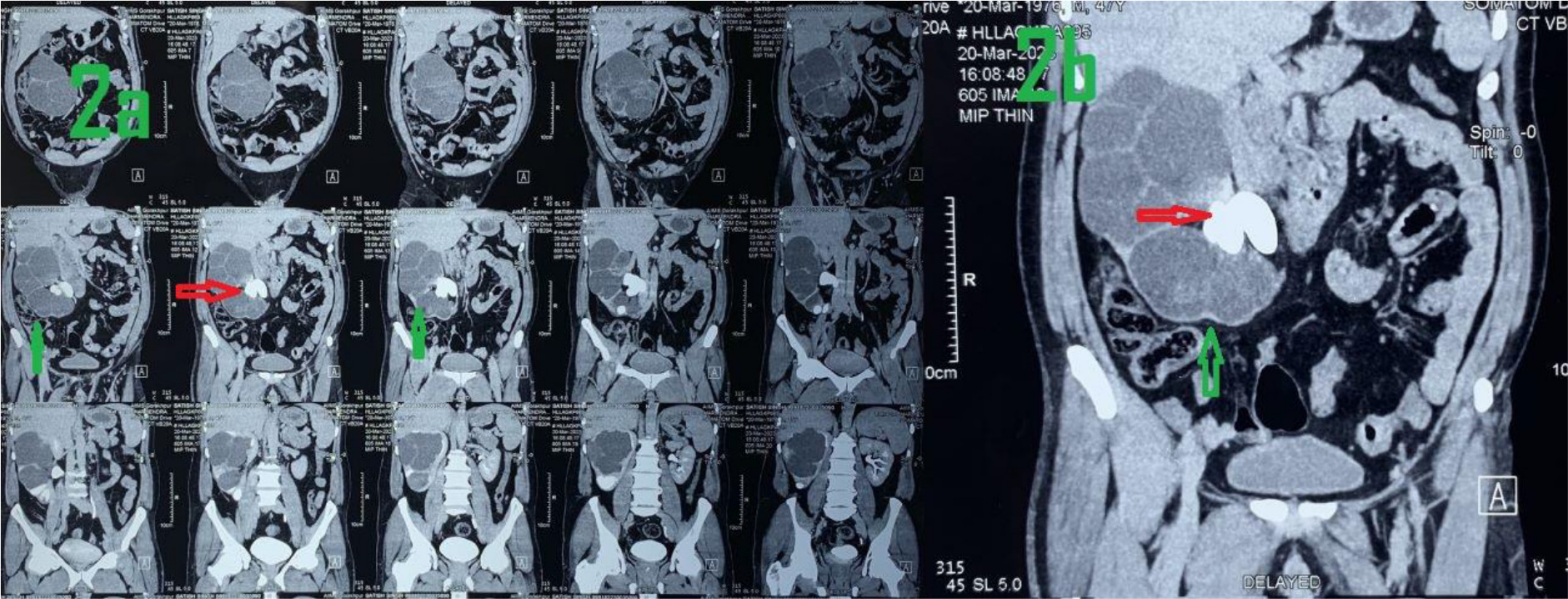
(A and B) NCCT showing large stone and lobulated kidney, depicted by red and green arrows, respectively.

A 47-year-old male attended our surgery outpatient department of All India Institute of Medical Sciences, Gorakhpur, India, with a history of right-sided colicky pain over the previous two months. Analgesics were able to provide relief, and there was neither fever nor blood in the urine. The pain began abruptly, was only moderately severe at first, gradually increased, began in the groin, and was referred to the loin. During the general examination, neither pallor nor edema nor lymphadenopathy was present. During the systemic examination, the patient’s abdomen was non-tender, soft, and devoid of any palpable lump. Both renal angles on either side were apparent. The pulse rate was 80 beats per minute, the blood pressure was 134/86 mmHg, the respiratory rate was 14 breaths per minute, and the temperature was 36.7°C. His bowel and bladder habits were normal, and there was no history of chronic medical disease or operative intervention. As the patient was having pain, he was hospitalized with analgesics and broad-spectrum antibiotics. Various investigations were done to establish the cause of the pain. An ultrasound examination of the kidneys, ureters, and bladder (USS KUB) uncovered a massive SC that measured 8 × 4 cm at its greatest width and was in the right renal pelvis and extending to the upper ureter and calyxes. There was an additional calculus of a lesser size in the lower calyx. These observations were corroborated by a plain X-ray of the KUB (Figure 1). A non-contrast computed tomography (NCCT) of the abdomen was carried out to define the anatomical structures more precisely for preoperative planning (Figures 2A and B). His preoperative investigations were within normal limits. Serum urea was measured at 28 mg/dl, and creatinine was measured at 1.1 mg/dl. Urinalysis revealed a moderate increase in specific gravity (1.030), significant hematuria (3+), and a trace of protein. The urine culture was sterile.

**Figure 3.**
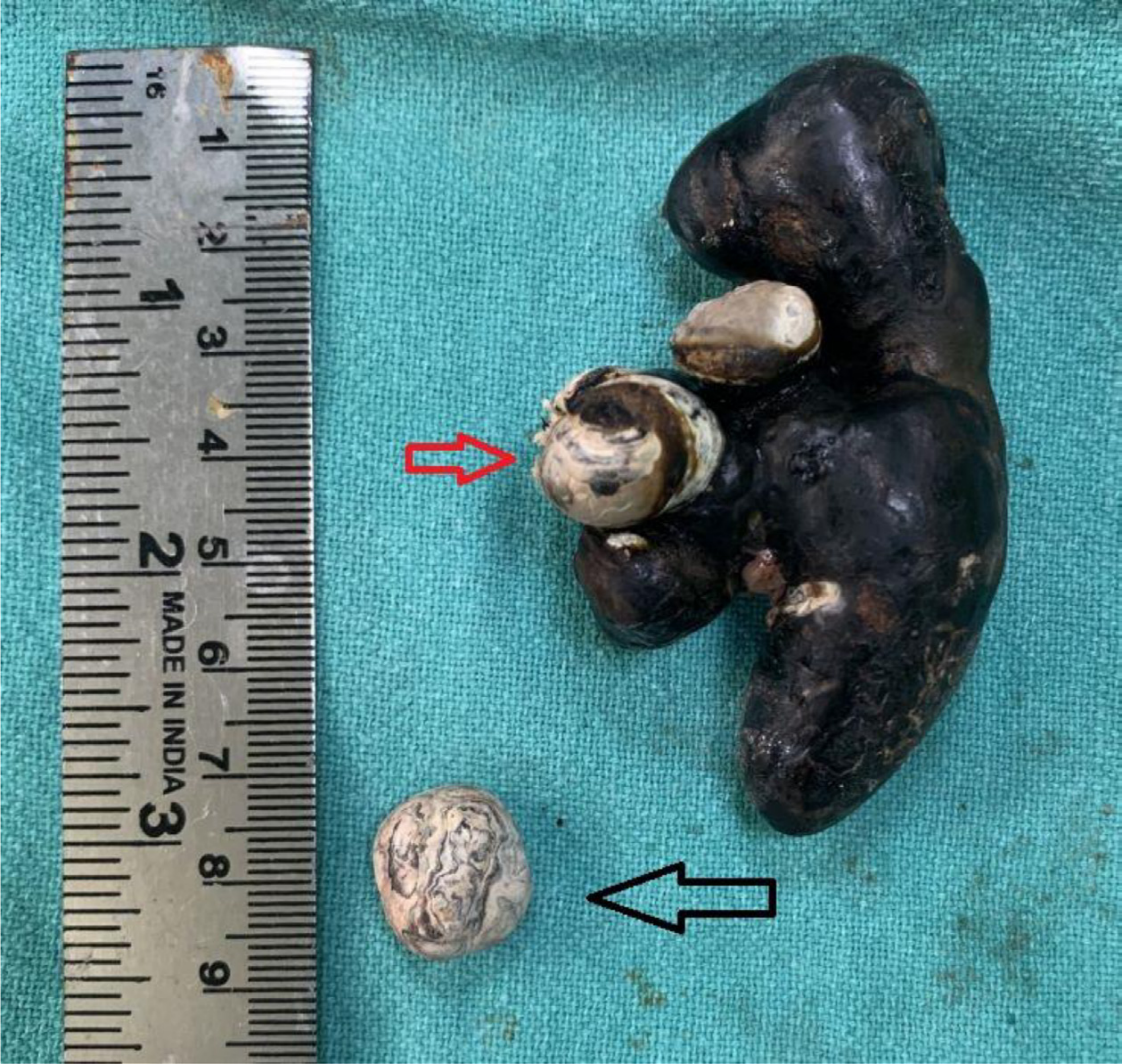
Huge staghorn calculus of approximately 8*4 cm in size with another small stone extracted from the renal pelvis and lower calyx, respectively.

Open pyelolithotomy was decided to be performed, and detailed informed consent for the same was obtained from the patient. The SC and the smaller calculi were extracted unfragmented (Figure 3). The patient was advised for a regular follow-up after 15 days, one month post-operatively, and every month thereafter for one year. The patient exhibited no symptoms, and an ultrasound examination revealed no signs of any leftover calculi. After taking written informed consent for publication from the patient, we prepare the case report for publication.

## Discussion

The lifetime recurrence rate for UC is between 10% and 75%, and the worldwide frequency is 14%.^7^ Notably, 1%-5% of the population is affected in Asia.^8^ 12% of the Indian population suffers from upper urinary tract urolithiasis.^7,8^ Initially, 10%-15% of all UC worldwide consisted of SC. Early and effective management strategies have reduced the current rate by 4% (6%-11%), primarily in developed countries. UC typically affects more men than women, whereas SC affects typically more women and is unilateral.^9,10^ Ultrasonography KUB has a sensitivity of 45% and a specificity of 94% when used to diagnose renal and ureteric calculi, respectively.^8^ It is the best first line of diagnosis because it is cheap and does not expose patients to radiation.^9^ According to the European Association of Urology (EAU) recommendations, an NCCT of the abdomen should be performed on patients with acute flank pain or suspicion of ureteric stones. The diameter and density of calculi are just two examples of the additional data they can provide for perioperative planning.^8^ The ultrasound, X-ray, renal scintigraphy, and NCCT were all a part of our diagnostic workup. Evaluation of UC relies heavily on markers of renal function, such as serum electrolyte and creatinine values. Renal scintigraphy may be necessary for further quantitative evaluation of large SCs.^11,12^

Nephrectomy should be contemplated for patients with negligible renal function (10%). SC has been linked to UTIs in 49-68% of individuals.^10^ *Proteus mirabilis, Klebsiella pneumonia, Pseudomonas aeruginosa*, and *Enterobacter* are the most found urease-producing microbes.^8,10^ Preoperative urine culture results are equal to antimicrobial prophylaxis. The bladder can be aspirated or catheterized sterilely to collect samples before surgery.^6^ This patient did not have a history of UTIs, and his urine culture revealed no bacteria or other organisms.

There is a 28% 10-year mortality rate and a 36% probability of developing renal impairment when SC is treated conservatively.^10^ As a result, surgical interventions such as PCNL monotherapy, ESWL monotherapy, a combination of PCNL and ESWL, and open and minimally invasive surgery are preferred. However, PCNL is still suitable for most cases, but laparoscopic pyelolithotomy can be used as alternative management in selected cases that are difficult to treat by PCNL.^13^ Renal failure often develops with SCs. Therefore, while carefully evaluating the stone’s location and size, the patient’s preferences, and the institutional capacity, adopting an aggressive therapeutic strategy is essential.^14^

In the resource-limited setting or the centers where minimal invasive technique is not available or if present but not easily affordable by the patients, an open pyelolithotomy is still an impervious approach to removing renal stones when a retro-renal colon (RRC) exists to avoid colonic injury during PCNL.^15^ PCNL is the preferred option for calculi size >20 mm, according to the EAU and American Urological Association, with a calculi-free outcome approximately on par with OSS.^16^ It has several benefits over OSS, including a lower risk of complications, a quicker recovery period, and a shorter hospital stay.^17^ Patients who should be considered for OSS or laparoscopy stone removal include those with non-functioning kidneys (totally or partially), anatomical malformations, and renal ectopia. Nephrolithotomy, ureterolithotomy, and cystolithotomy may be required in exceptional circumstances. According to Chen et al. in their systemic review and meta-analysis of 10 studies with 921 patients,^5^ standard PCNL appears to be a secure and useful alternative to OSS or mini-PCNL for patients with SC, with many advantages including a shorter hospital stay with early resumption of activities, a shorter operative duration, less blood loss and need for blood transfusion, and fewer surgical complications. They recommended conducting additional large-sample perspectives, multi-centric studies, and randomized control trials (RCTs) to support their findings. As it allows for the simultaneous restoration of renal collecting system anatomical abnormalities, OSS had a better calculi-free status in instances with numerous calculi with calyceal expansions. This patient had a huge SC in the right kidney with additional calculi in the lower calyx. Given the complexity of the case, it was decided that a right-sided open pyelolithotomy would be the best treatment. Both the operation and subsequent recovery went smoothly. There were no calculi at the 1-month follow-up.

## Conclusion

The use of PCNL is the recommended treatment for SC. On the contrary, huge and complex SCs accompanied by other or multiple calculi in the immediate vicinity with significant renal functional impairment are most effectively treated with OSS. This method achieves optimal outcomes through a singular process, obviating the necessity for multiple steps, such as PCNL, to remove calculi. Therefore, the OSS should continue to be taught to medical students and put into practice whenever necessary because it still applies to modern urological practice.

## Availability of Data and Material

It is a case report, and all data, including images, are being submitted in the case report itself.

## Authors Contributions

DP, SJ, and PB analyzed and interpreted the patient data, performed the surgery, collected the imaging, and wrote the manuscript.

## Consent for Publication

Informed written consent was obtained from the patient to publish this report and the accompanying images.

## Competing Interests

The authors declare that there are no competing interests.

## Ethical Approval

Ethics approval and consent to participate are not applicable as it is a single case report, not a case series.
